# Preventive home visits and health – experiences among very old people

**DOI:** 10.1186/1471-2458-13-378

**Published:** 2013-04-23

**Authors:** Lina Behm, Synneve Dahlin Ivanoff, Lena Zidén

**Affiliations:** 1 Sahlgrenska Academy, Institute of Neuroscience and Physiology, Department of Clinical Neuroscience and Rehabilitation, University of Gothenburg, Box 455, 405 30 Gothenburg, Sweden; 2 Vårdalinstitutet. The Swedish Institute for Health Sciences, Lund University, Lund, Sweden

**Keywords:** Aged, 80 and over, Health promotion, Disease prevention, Multi-professional, Preventive home visit

## Abstract

**Background:**

As more people reach older age, there is a growing interest in improving old person’s health, activity, independence and social participation, thereby adding quality to the extended years. Preventive home visits (PHV) programs for old people have received much attention in recent decades. A large body of research shows mixed effects, and argues that a home visit is a complex social process influenced by numerous factors. To evaluate the impact of PHV, as well as making decisions on whether, how, and to whom the service should be provided, requires a deeper understanding of PHV than we have now. Consequently, the aim of the study was to describe the variations in older people’s (80+) experiences of a single preventive home visit and its consequences for health.

**Methods:**

Seventeen participants between 80 and 92 years of age who had all received a structured PHV were interviewed in their own homes. The interviews were analyzed using the phenomenographic method, looking at the variations in the participants’ experiences.

**Results:**

The interviews revealed four categories: “The PHV made me visible and proved my human value”; “The PHV brought a feeling of security”; “The PHV gave an incentive to action”; and “The PHV was not for me”.

**Conclusions:**

The experiences of a PHV were twofold. On one hand, the positive experiences indicate that one structured PHV was able to empower the participants and strengthen their self-esteem, making them feel in control over their situation and more aware of the importance of keeping several steps ahead. Together this could motivate them to take measures and engage in health-promoting activities. On the other hand, the PHV was experienced as being of no value by a few. These findings may partly explain the positive results from PHV interventions and emphasize that one challenge for health care professionals is to motivate older people who are healthy and independent to engage in health-promoting and disease-preventive activities.

## Background

The rapid rise in the number of very old people (80+) in Sweden and elsewhere represents a major challenge with regard to both the quality and the costs of health care [1,2]. This has led to a growing interest in improving old people’s health, activity, independence and social participation, thereby adding quality to the extended years. The interventions that have shown the most promising effects in very old people integrate both health-promotion and disease-prevention [3]. For this reason, a health-promoting, disease-preventive, multi-dimensional and multi-professional intervention, called” Elderly persons in the risk zone”, were set up, to evaluate and compare the effects of group education and those of preventive home visits (PHV) [4]. The study addressed very old people living in their own homes that were close to developing frailty (“pre-frail”). As both interventions were complex, qualitative methods were required to capture their multi-dimensionality. This study addresses those who received a structured PHV. If respondents are given the opportunity to highlight their own individual priorities, it might be possible for those working with health-promoting and disease-preventive interventions to improve their understanding of the impact of the intervention [5].

Very old people are often described as a frail and vulnerable group [6]. Nevertheless, a large proportion of these people over 80 years are in good health and live in their own homes, managing most of their daily activities on their own [7]. These persons who are independent of help from others tend to report fewer symptoms and higher quality of life than those who are dependent on help [8]. It has been reported that very old people who are in a “pre-frail” phase are the ones who benefit most from preventive interventions [9]. As many older people are physically active and have a strong inner drive to maintain health, they should be a suitable target group for health- promoting and disease-preventive interventions [10].

Preventive home visit (PHV) programs for old people have received much attention in recent decades. The general aims of such programs are to maintain health and independence, as well as preventing disability and hospital care, thereby reducing costs [4,11,12]. However, the value of PHV has been discussed in several reviews, as effects are mixed and difficult to compare [13-15]. Positive effects have been found on for instance mortality, nursing home admissions, falls, functional decline and ADL [14,16-19]. While others argue that there are no clear evidence in favor of the PHV [13]. In the study” Elderly persons in the risk zone”, Gustafsson *et al.* found positive effects on self-rated health three months after intervention [20] and ADL dependence for up to two years [21]. Ingredients for success have proven to be, for instance, if the PHV involves older people in an early and reversible phase of poor health or disability [9] and adopts a multi-professional approach [22,23]. The duration of the PHV, i.e. a larger number of visits, is also associated with more positive effects [15]. Despite the outcome, the participants in PHV programs have generally experienced the PHV as positive, since they were given the opportunity to discuss problems with professionals and received attention and support, which made them feel more secure [9,24,25].

A PHV is described as a complex social process influenced by numerous factors [26]. Thus, to evaluate the impact of PHV, as well as making decisions on whether, how, and to whom the service should be provided, requires a deeper understanding of PHV than we have now [27]. Studies focused on the users’ perspective of interventions are rare, and despite the large number of reports on PHV interventions, studies that specifically elucidate how the very old experience the PHV are lacking.

The aim of the present study was to describe the variations in older people’s (80+) experiences of a single preventive home visit and its consequences for health.

## Methods

### Participants

A total of 17 individuals, 12 women and five men, aged 80 to 92, were recruited from the larger intervention study “Elderly persons in the risk zone” in which they had all received a structured preventive home visit (Table 1). The inclusion criteria for this study were being 80 years or older, living at home, being independent of help from others and being cognitively intact.

**Table 1:** Socio-demographic and characteristics of the interviewed participants

**Interview person**	**Age**	**Gender**	**Living conditions**	**Social status**	**Perceived health**
1	92	Man	Apartment	Living together	Excellent
2	87	Woman	Apartment	Living alone	Reasonable
3	90	Woman	Apartment	Living together	Excellent
4	89	Woman	Apartment	Living together	Reasonable
5	87	Man	House	Living together	Good
6	88	Man	Apartment	Living together	Reasonable
7	81	Woman	House	Living alone	Reasonable
8	80	Woman	Apartment	Living alone	Good
9	80	Woman	House	Living alone	Very Good
10	83	Woman	Apartment	Living together	Very good
11	80	Woman	House	Living together	Good
12	85	Woman	House	Living alone	Good
13	83	Woman	House	Living together	Good
14	87	Man	House	Living together	Reasonable
15	80	Woman	House	Living together	Very good
16	82	Man	House	Living together	Very good
17	80	Woman	Apartment	Living alone	Good

The PHV aimed at establishing contact and discovering questions, as well as identifying unmet needs that could be met by the municipality or voluntary associations. The PHV took the form of a single home visit made by either a nurse, a physiotherapist, a qualified social worker or an occupational therapist. During the PHV the clients received verbal and written information and advice about what the municipality could provide in the form of local meeting places, activities run by local associations, physical training for seniors, walking groups etc. Information was also given about the various kinds of help and support offered either by volunteers or by professionals, and accessibility to assistive devices and housing modifications. In addition, environmental fall risks in the home were identified, and advice on how to prevent them was included. Information was also given about whom the older person could contact for different problems. The preventive home visit was guided by a protocol which included an opportunity for individuals to further elaborate on certain elements (Table 2). The PHV lasted between one and a half to two hours. If the participant raised a question outside the purview of the attending professional, he/she was informed where to turn to in order to receive comprehensive information [4].

**Table 2:** The elements in the protocol used in the preventive home visit

**No**	**Protocol elements**
1	Information and advice about, and when appropriate instructions in, a basic home exercise program including balance exercises
2	Assessment of the fall prevention checklist, information and advice on how to prevent identified fall risks and continue be active, and in adequate cases a “safety walk” in the home
3	Information and advice about technical aids and housing modifications, and, if necessary, where and whom to turn to for purchase or application
4	Information and advice about smoking alarms, and, if necessary, an offer to check the smoking alarm
5	Information about the range of help and support available in Gothenburg and in the municipality (volunteers, churches, mission fellow human, health centers etc.), and where to turn to for help with health problems and illness, opening hours, phone times, and phone numbers
6	Information on the possibility of an appointment with a pharmacist at the local pharmacy for review of and counselling on medicines
7	Information and advice about incontinence
8	Display and hand over a brochure with information on the Swedish legislation and possibilities for advise on and assessment of driving capacity by professionals
9	Information and advice about what the municipality can provide in the form of local meeting places, activities run by local associations, physical training for seniors, walking groups for seniors, and possibility of receiving or providing volunteer interventions
10	Offer to register for “try-out” activities, a standalone group visit to local meeting places, a short introduction to computer sciences, petanque clubs for seniors, gyms for seniors, Nordic walking groups, etc
11	Information about public transportation, including busses adapted for older adults, and of mobility service for the disabled
12	Information on the Social Services Act, and on where and whom to contact in the municipality in order to apply for home care services

### Procedure

In accordance with the phenomenographic tradition, the subjects were chosen strategically in order to represent as many aspects of experiences of a PHV as possible. Thus, a purposeful selection of individuals with different background such as marital status, living conditions, age, and perceived health was made. The participants were recruited one by one consecutively over a period of 6 months, and our aim was to include a total of 15 to 18 persons. In total 48 participants received an information letter about the study directly after the PHV. Those who matched our selection criteria and had agreed to participate, giving their written consent, were contacted by the researcher and the interviews were booked (n=17). The interviews took place two to three weeks after the PHV. The interviewer (first author) was a registered public health nurse and had not been involved in performing the visits. To create an informal interview situation, the interviews were carried out in the participant’s own home. The interviews started with a few minutes ‘small talk’ and information about the procedure. The interviews were semi-structured, and as an introduction the participants were asked to indicate how they experienced their current health by selecting one of the alternatives on a 5-point scale: “excellent health”, “very good” “good” “reasonable” or “bad”. The reason for asking this question was to encourage the participants to start reflecting upon their health, as well as obtaining a more detailed description of the sample. They were then asked to describe their experiences of the home visit and the consequences for their present and future health. Follow-up questions and prompts were used, such as ‘Tell me more about that’ or ‘What does this mean to you?’ and ‘Can you clarify?’ The ambition was to let the participants concretize their experiences as much as possible.

The Regional Ethical Review Board in Gothenburg, Sweden, approved the study (650–07).

### Analysis

The interviews were recorded and lasted for an average of 36 minutes (range 26–60). The first author (LB) carried out the interviews and transcribed them verbatim. All interviews were transcribed before the start of the analysis process. The analysis was based on the phenomenographic method [28] described by Dahlgren and Fallsberg [29], and comprised the following steps:

1. All the interviews were first read thoroughly and repeatedly to obtain a total concurrent overview, a sort of familiarization.

2. The second step, condensation, was a selection procedure. Qualitative meaning quotes that dealt with the experience of the preventive home visit were extracted from all interviews to achieve a concentrated and representative version of entire dialogues. The quotes thus selected made up a pool that formed the basis for the following steps in the analysis.

3. The third step, comparison, was to contrast the extracted quotes with each other in order to uncover sources of variation or agreement. In the grouping step, similar quotes were grouped together.

4. The next step, articulating, was an attempt to describe the essence of the similarity within each group.

5. The labelling step gave the categories names that corresponded to the essence of their meaning.

6. The last step, contrasting*,* compared categories with each other to arrive at a definitive description of the unique character of each category. In this final step the various descriptions dealt with in the categories were defined and named, summarizing the common significant meaning in each category.

There was a constant interplay in the entire process between the various steps of the analysis. The ambition was to ensure that each category was qualitatively unique, that they did not overlap, and that there was empirical support for each category. As the focus here is not on the subjects but on the qualitative meaning of each category, the categories are mutually exclusive, and each interviewee can belong to more than one category. The whole sequence of steps in the analysis was followed separately by the first (LB) and second author (LZ) before joint discussions leading to consensus. Finally, the third author (S D-I) listened to all the recorded material and validated the analysis. In the validation process the authors compared their findings for similarities and differences until agreement was reached.

## Results

The interviews revealed that the PHV could lead to positive changes, such as accentuating the participant’s human value, bringing a feeling of security and giving an incentive to action. On the other hand, a few of the participants found the PHV difficult to assimilate, either because they felt too ill or because they felt that they could manage on their own. The analysis of the interview data resulted in four categories: 1. The PHV made me visible and proved my human value: 2. The PHV brought a feeling of security: 3. The PHV gave an incentive to action; 4. The PHV was not for me.

The experiences expressed were multidimensional, and most of the interviewed participants expressed experiences that belonged to more than one category (Table 3).

**Table 3:** Allocation of the 17 participants into the four categories

**Category**	**1**	**2**	**3**	**4**	**5**	**6**	**7**	**8**	**9**	**10**	**11**	**12**	**13**	**14**	**15**	**16**	**17**	**Total**
The PHV made me visible and proved my human value			X				X	X	X	X	X		X				X	8
The PHV brought a feeling of security	X	X	X	X	X	X	X	X	X	X	X	X	X		X	X	X	16
The PHV gave an incentive to action	X	X	X	X	X	X		X	X		X	X			X	X	X	13
The PHV was not for me												X		X			X	3

In the following presentation of the findings, the categories are illustrated by quotations from interviews with the different participants. To make reading easier, the interview texts have been edited to some extent; dots (. . .) mark words or parts of the conversation that are left out and any text within [] is a clarification made by the authors.

### The PHV made me visible and proved my human value

In this category the participants expressed that the PHV had proved their value as human beings despite their age, and they stressed how important it was that someone was interested in the thoughts and needs of an older person. The very fact that somebody spent time with them was an essential part of the value of the PHV. Earlier participants of this category had experienced being viewed as insignificant, even invisible, both in social and healthcare settings. Nobody seemed to be genuinely interested in how they managed and how their health status was. They attached great importance to the fact that someone now seemed to care about older people and that resources were being allocated to enhance their health. This made them feel that they were still important members of society, that they were not abandoned after all, and that their status as valuable human beings was confirmed.

(Interview 13):

P: Just that they think about us, it's nice, they think of older people. And provide information about options, where you can get help … There are many lonely people, having no relatives…

I: Yes, does it have some significance to you personally, that you feel that they're thinking about older people?

P: Yes, it’s clear that it has. It's good. The fact that you are visible, so to speak. That you are still here. So they’re not only talking.

I: What do you mean?

P: Politicians, they talk and talk about how they are going to make it better for older people, but it never happens.

### The PHV brought a feeling of security

Within this category the participants expressed that being aware of whom and where to turn to when they needed help brought a sense of security. The participants valued the information they received at the PHV, and they had saved the brochures for future use in case of need. They also appreciated that the brochures had been given to them personally rather than being sent by mail. They expressed that it was easier to absorb the information as they could discuss it face to face, which also made it easier to understand and integrate the information. Actually meeting a person whom they could contact later also gave them a sense of security.

(Interview 16)

I: What was most important for you concerning the PHV?

P: I must say it was the contact with the visitor, and that we met face to face, that we not only got a piece of paper home, it meant a lot to us, that we got that knowledge and that it was done this way…

I: Why does it matter that it was done this way?

P: Well, I think you absorb it better, you understand it better, what's available. Otherwise I think that we would have just thrown away the brochures and thought that we would wait to deal with it until something happens. Now we know about this, we have received a visit, it remains in our memory.

The participants felt that they had received new information, for instance, what kind of help was available or how to avoid accidents. They also experienced that they had received information and suggestions about various things that had later turned out to be helpful in their daily lives, for example, practical help with snow removal in winter or changing light bulbs or putting up curtains, which could be hazardous for an older person.

(Interview 4)

P: The information I received has opened doors that I did not know about and that is helpful. Most significantly, the help we have received in the home here, it has been a tremendous help to us … and think of all the old people who fall and break their bones when they stand on chairs and balances, but what can they do? But we don’t need to think about that; we have received help so we never have to do it.

The PHV could also help the participants to get assistive devices, for instance, a shower stool, which made them feel safer in daily activities. The participants said that the PHV could decrease feelings of worry, thus increasing their chances of remaining in their own homes. Altogether this gave the participants a feeling of security.

### The PHV gave an incentive to action

This category contains the experience that the PHV motivated the participants to start thinking about how to keep several steps ahead, so to speak, to be able to prevent future health risks and illness. For instance, they considered performing various health promotion activities aiming at prevention of ailments that could come with age. Thus the PHV had evoked an awareness of a possibility to influence future health. The participants described the importance of learning new ways to look at situations and trying new things, not getting stuck in old habits and patterns. The PHV provided practical information on how to strengthen physical functions, or how to prevent future functional losses. Experiencing the body in a positive way and being assured that it was possible to influence it was thus experienced as very helpful, especially as the general view in society is that age primarily means negative bodily changes. The incentive to action thus included a new and more positive way of looking at the body.

(Interview 11)

P: Now you are old, but look how much you can do, and it's me who will do it. It’s not them, it's me who will do all the things they talked about. I need to engage in all these activities, I cannot just sit. … I have an insight, an insight into everything that I can do now and that feels very important.

The participants felt that the PHV had generated motivation to get out of the home and start activities such as gymnastics and exercise or to visit different meeting points or cultural activities. Participants within this category also experienced that the PHV had provided them with inspiration to think in different ways about what was important in life, to take care of themselves and to prioritize things differently.

### The PHV was not for me

The participants within this category expressed that the preventive home visit was nothing for them; they either experienced it as too”demanding” because they had symptoms that hindered them, or they did not consider that they needed the resources offered by the professional visitor because they were too healthy.

Some of the participants in this category were not ready for interventions or information concerning anticipated future needs. They said that before the PHV they had already adapted to an experienced, or expected, decrease in physical capacity in various ways, such as giving up bicycling, getting Nordic poles, getting private help with window cleaning or with snow clearing. They also thought that as long as they were independent of help from others and could manage in daily life they were not interested in the information offered at the PHV. The participants were proud of being able to manage on their own, It gave them a sense of control over their situation; being active helped them to stay alert.

(Interview 17)

P: As long as you have a clear head, I won’t admit that I am that old, I won’t.... It ‘s different if you are walking a bit handicapped and you can feel it if you can’t manage it. As long as I don’t feel it I won’t admit that I’m as old as that. It’s strange, but that’s the way it is.... As soon as you can’t walk, you have to walk more slowly, and then you will admit it [that you are old]. But… if you don’t feel that you’re old then you can keep up rather well and so on. Well, you don’t run along on ladders and balance and so on, like you did when you were younger, that you can’t do. As long as we can manage ourselves, I mean, that is what keeps us vigorous and alert.

The participants also said they did not worry in advance, and that they could search for and obtain information when they felt that they needed it. They were content with life and expressed that hopefully they would never need the information and help that was offered by the municipal health care service.

In contrast to this experience, some of the participants in this category expressed hopelessness and a lack of trust in the future. These participants described a succession of functional losses, such as impaired hearing or walking ability, which had restricted their daily lives. The PHV was considered to be of no importance because they felt too ill. The participants described experiences of being let down, that their lives had changed dramatically and become more restricted both physically and socially.

(Interview 14)

I: I wonder how did you experience the PHV?

P: Well, I don’t know if it has made any difference… I mean, I have had no joy of it at all… That thing, that you should go out and meet other people and so on, that’s nothing for me. It’s very difficult for me to attend when there are many people [because of difficulty of hearing]… I just spoil it for them… I guess I am like an ordinary 85-year-old, not much more to expect.... it goes as long as it goes.

## Discussion

The findings throw light on older people’ experiences of a preventive home visit and its consequences and may help us to gain a deeper understanding of the intervention. It also generates a discussion about to whom the PHV should be offered and gives us a better understanding of the results from the study “Elderly persons in the risk zone”, which showed positive results for perceived health and ADL [20,21].

Our findings show that a preventive home visit may accentuate the human value of older people, thus making them feel still valuable though vulnerable. Our Findings confirm the theory of Henriksen and Vass [30], who suggest that one reason for the positive results of a preventive home visit may be that the older person is taken seriously and participate in decisions concerning his/her own health. The feelings described above, of being invisible in society, that no one is genuinely interested in older people, how they managed or about their health status, may be seen as signs of ageism [31]. Our interviews revealed that knowing that someone is interested, listens and takes time to talk to them about important things makes older people feel valuable despite their age. To strengthen the older person’s self-esteem and to empower them is an important part of all health-promoting interventions [32]. Empowerment means giving responsibility and a chance to participate to another person, while taking a step backwards, and to indicate, encourage, and inform [33]. It is a common view that people who have a high degree of confidence concerning their own health, or a high degree of self-efficacy, are more inclined to engage in health-promoting activities than those who have low degree of self-efficacy [34]. Theander and Edberg [12] claim that the benefits of preventive home visits are twofold; on the one hand, it may strengthen older people and increase their self-efficacy, while, on the other hand, it supplies the professionals with information about the individual’s health, concerns and life situation. Thus, the strengthened self-esteem may result in the participants engaging in health-promoting activities.

Our Findings also show that information about health care, where to turn to when needing help and what help was available, could give the visited person a sense of security. Feeling secure may be based on a feeling of control over the situation, which can be called locus of control [35]. Hendriksen and Vass [30] suggest that if older people are provided with information about health care, it may increase their ability to use their own resources to take measures. Theander and Edberg [12] point out that, in order to make the exchange of information between the older person and the visitor to work, it is important that the older person feels secure with the visitor and that the visitor has sufficient knowledge of health promotion. Yamada and coworkers [36] believe that the visitor’s competence is one of the most important factors in reaching a positive result, i.e. that the visited persons feel that they may benefit from the PHV. Other studies have shown that the components of the preventive home visit may affect the outcome. A multidisciplinary approach [22,23] and a structured dialogue between the visitor and the older person are said to be essential factors in ensuring a successful outcome [37]. In this study the PHV that the participants received was structured and the content was multi-dimensional. The PHV was made by either an occupational therapist, physiotherapist, nurse, or a social worker, which jointly planned the PHV. Thus, the PHV could be described as multi-professional as it was based on a holistic approach to health, where knowledge from different professions was brought together. This approach is vital in health-promotion and disease-prevention for older people as no single approach has been found to prevent the complexity of the deterioration that comes with advancing age [14,15,38].

The participants described that the preventive home visit had generated motivation to start engaging in different health-promoting activities in order to prevent health problems that could arise later. Participants in the category “the PHV gave an incentive to action” experienced that they had become more aware of the importance of keeping several steps ahead. The Health Belief Model (HBM), which is a model developed to predict people’s health behavior [39], describes two different triggers which can produce actions by an individual: external triggers (such as advice, mass media or increased awareness) and internal triggers (illness or physical symptoms). The PHV could work as a trigger through the information that the participants receive, as well as by empowering the participants to take control over their own life situation. In contrast to general health care, the PHV focused on health aspects, inspiring the older people to take their own health promoting-actions. It is therefore possible that the PHV may help older people to acquire a positive view of their own ageing and encourage them to take a more positive view of the future and of their own ability to influence health. There is however a distinction between the participants’ experiencing the PHV as an incentive to action and it actually resulting in action. Published results from” Elderly persons in the risk zone” showed that the PHV delayed deterioration in ADL for up to two years [20,21]. This could be seen as evidence that the PHV did activate most of the participants.

Taken together these positive Findings may partly explain the positive results that have been shown in quantitative studies of PHV. The PHV empowered the participants, strengthened their self-esteem, it gave them information that made them feel in control and it also got them more aware of the importance of keeping several steps ahead. This could have increased the participant’s ability to use their own resources and may have motivated them to take measures and engage in health-promoting activities.

In contrast to the reported positive effects of the PHV, we found that some of the participants declared that “the PHV was not for me”. They said that the PHV and the information offered by the visitor were not important or relevant at that moment. Nilsson and coworkers [40] described that older people often live in the present. This may be due to fear or insecurity concerning expected negative changes that may threaten health during ageing. A recent focus group study reported that older people who live in the present thought that it was difficult to assimilate information that they did not consider to be of current interest [41]. The fact that older people live in the present and find it difficult to assimilate the information might also explain the finding mentioned above. In another study, Sahlen [42] reported that participants in a preventive home visit felt that they did not need the PHV and that the PHV came too early. However, it has been shown that interventions targeting older people in an early or reversible stage of frailty have led to more positive results than those targeting older people whose functional decline is more advanced [36]. Hence, one challenge for health care professionals is to motivate older people who are healthy and independent to engage in health-promoting and disease-preventive activities. As older people value and strive for independence [43] and control [44] in daily life, one possible motivating reason to participate in health-promoting activities is to support and maintain independence. Health care professionals could use this motivator in their promotion work among older people.

Something that further leads us to believe that it is important that the PVH comes at an early or reversible stage of frailty is that some of the participants in our target group of independently living older people expressed that the PHV was nothing for them because they felt too ill. We therefore have to be aware of the fact that independent living does not mean that these old people were healthy as living longer in most cases also means having more symptoms and diseases [45]. It is thus evident that the participants in the category “the PHV was not for me” did not regard themselves as a target group for PHV interventions. It is possible that interventions targeting older people may need to be individually tailored to meet each person’s needs at that moment. Against this background it seems important that health care professionals who design health- promoting interventions should learn more about the older people’ situation, their own views of what influences health, what interventions and information they need and when they consider it to be the right time for a visit. The findings of our study provide new information and could thus act as an important piece in the larger jigsaw puzzle that challenges all professionals concerned with setting up health-promoting interventions targeting older people.

The aim of our study was to explore the participants’ different experiences of a preventive home visit and its consequences for perceived and anticipated health in order to form a picture of the spectrum of these experiences. The fundamental assumption underlying the phenomenographic approach is that a qualitative variation exists in how people experience phenomena [46]. Its aims are to reveal this variation in experiences and describe it in categories, in other words, to describe the world as people see it [46]. The method chosen served the purpose in a satisfactory way as a means of gaining access to the participants’ various experiences. A limitation was that we were able to include only five men in our study. Another limitation was that we only obtained participants who reported reasonable, good or excellent health in the introduction of the interview, while no participant reporting bad health agreed to participate. However, as this study addressed home-dwelling cognitively intact people who managed their daily activities on their own, the ones with bad health were not our target group.

There are some further limitations to the study. One limitation was difficulty in reaching the second order of the participants’ experiences of the PHV in some of the interviews. In phenomenography the second order is central [28]. This means that the focus of the interviews is to capture the participant’s different ways of thinking rather than giving a superficial description of the phenomenon studied, in this case the PHV. In our interviews, the degree to which the participants wanted to open themselves in the interview situation varied, and in one or two of the interviews they chose to describe the content of the home visit and to show the brochures they had received rather than talking about their own thoughts and experiences. This meant a challenge to the interviewer. On the other hand, other participants described their experience of the PHV and its consequences in great detail. However, we cannot exclude the possibility that there may be other ways of experiencing the PHV and its consequences than were reported in this manuscript. Nevertheless, the variation of experiences that emerged from the interviews did create an outcome space covering qualitatively different aspects of the studied phenomenon, which was the aim of the study. However, it is possible that the findings would be different if the participants had participated in a program with more than one PHV or if the PHV had contained more dimensions.

The experienced consequences of the PHV were multidimensional, and most of the interviewed subjects belonged to more than one category. There was thus variation between the participants, as well as within each subject, in the way that they experienced the PHV. Two subjects belonged to the category The PHV was not for me, as well as to the categories The PHV gave an incentive to action and The PHV brought a feeling of security, which may appear contradictory. However, this can be seen as examples of how complex the experiences were and can be understood as a qualitative variation in ways of seeing and acting in different life situations.

Another risk of bias that needs to be addressed is subjectivity. A common question in qualitative analysis is how much the researcher is coloured by preconceptions. No method is neutral, and it is very difficult to disregard preconceived ideas about a specific phenomenon. One way to enhance the trustworthiness and reliability of the findings is to illustrate the categories with quotations from the interviews [47]. Another way is to test the trustworthiness of the descriptive categories by adding an independent assessor [48]. In the present study the authors first read and analysed the interviews separately and then reflected and discussed together until a consensus was reached on the category description. Nevertheless, the validity of an interview study is always questionable, since there is no way of ensuring that the participants really share their profound experiences with us or that the interpretation of what has been said is correct. However, during the interviews, the participants or the interviewer were able to return to the topic, which may strengthen the validity of the findings. In phenomenography, the researcher processes the verbal expressions in the interviews in order to understand the personal meaning intended by the participants [28]. In the analysis, and in describing the findings, we have tried to stay as close to the participants’ statements as possible. The authors were all of different professional backgrounds, a public health nurse, a physiotherapist and an occupational therapist. Two of them conducted the analysis of the interviews, while the third verified the analysis after listening to all the material. This might strengthen the objectivity i.e. the investigator's ability to be neutral and not colour the data with their own opinions and attitudes [48].

Finally, generalizability needs to be addressed. In qualitative studies this is not the goal, rather the aim is to be able to transfer the findings to other similar contexts [49]. Nevertheless, since the findings of qualitative studies are often context-dependent, it is important to reflect upon how transferable these findings are. We believe that the combination of qualitative and quantitative methods could maximize the ability to bring different strengths together and provide a unique opportunity to see what is in the "black box", i.e. to generate unexpected or unpredictable knowledge. Further qualitative and quantitative studies are therefore needed to provide a broad picture of the intervention preventive home visit.

## Conclusions

This study reports that a preventive home visit can be experienced in different ways, depending on the visited person’s situation. On one hand the positive experiences of the PHV indicate that a single, well-structured preventive home visit is able to empower the participants, strengthened their self-esteem, give them information that makes them feel in control and get them more aware of the importance of keeping several steps ahead. Together this could increase the participant’s ability to use their own resources and motivate them to take measures and engage in health-promoting activities. On the other hand, the PHV was experienced as being of no value by a few, either because they felt too ill or because they did not feel ready for the information.

The findings in this study could partly explain the positive results from quantitative studies of PHV interventions and emphasize that one challenge for health care professionals is to motivate older people who are healthy and independent to engage in health-promoting and disease-preventive activities.

## Competing interests

The authors declare that they have no competing interests.

## Authors’ contributions

LB and LZ were responsible for the study design. LB collected the data and was the primary author of the paper. LB, LZ and SD-I performed the data analysis. All of the authors contributed to writing and reviewing the manuscript. All authors read and approved the final manuscript.

## Pre-publication history

The pre-publication history for this paper can be accessed here:

http://www.biomedcentral.com/1471-2458/13/378/prepub
